# Electric Double Layer Based Epidermal Electronics for Healthcare and Human-Machine Interface

**DOI:** 10.3390/bios13080787

**Published:** 2023-08-03

**Authors:** Yuan Gao, Hanchu Zhang, Bowen Song, Chun Zhao, Qifeng Lu

**Affiliations:** 1 School of CHIPS, XJTLU Entrepreneur College (Taicang), Xi’an Jiaotong-Liverpool University, 111 Taicang Avenue, Taicang 215488, China; yuan.gao2104@student.xjtlu.edu.cn (Y.G.); hanchu.zhang21@student.xjtlu.edu.cn (H.Z.); bowen.song21@student.xjtlu.edu.cn (B.S.); 2 School of Advanced Technology, Xi’an Jiaotong-Liverpool University, Suzhou 215123, China; chun.zhao@xjtlu.edu.cn

**Keywords:** epidermal electronics, flexible devices, electric double layer, physiological signal monitoring, healthcare, human-machine interface

## Abstract

Epidermal electronics, an emerging interdisciplinary field, is advancing the development of flexible devices that can seamlessly integrate with the skin. These devices, especially Electric Double Layer (EDL)-based sensors, overcome the limitations of conventional electronic devices, offering high sensitivity, rapid response, and excellent stability. Especially, Electric Double Layer (EDL)-based epidermal sensors show great potential in the application of wearable electronics to detect biological signals due to their high sensitivity, fast response, and excellent stability. The advantages can be attributed to the biocompatibility of the materials, the flexibility of the devices, and the large capacitance due to the EDL effect. Furthermore, we discuss the potential of EDL epidermal electronics as wearable sensors for health monitoring and wound healing. These devices can analyze various biofluids, offering real-time feedback on parameters like pH, temperature, glucose, lactate, and oxygen levels, which aids in accurate diagnosis and effective treatment. Beyond healthcare, we explore the role of EDL epidermal electronics in human-machine interaction, particularly their application in prosthetics and pressure-sensing robots. By mimicking the flexibility and sensitivity of human skin, these devices enhance the functionality and user experience of these systems. This review summarizes the latest advancements in EDL-based epidermal electronic devices, offering a perspective for future research in this rapidly evolving field.

## 1. Introduction

The skin, as the largest organ in the human body, serves as a critical interface for sensing environmental stimuli and extracting bioelectric signals [1]. This unique position renders it an invaluable platform for healthcare applications, disease diagnosis [2], and human-machine interaction. Consequently, the field of epidermal electronics, which focuses on the development of flexible and conformal electronic devices to monitor physiological signals from the human body, has experienced a surge of interest and growth [3].

However, conventional electronic devices often fall short in terms of flexibility [4], biocompatibility [5,6,7], and permeability [8], permeability refers to the ability of a device to allow for the natural exchange of substances, such as gases, fluids, or even ions, between the skin and the environment. This attribute is particularly important to maintain the skin’s normal functions and health, like breathing and sweat secretion, while the device is attached. Highly permeable devices can minimize the obstruction of these natural processes, reducing the potential for skin irritation or damage and improving the comfort and wearability of the device. which are essential for effective interfacing with the skin. This discrepancy leads to significant mechanical and biological mismatches [9,10]. Therefore, the pursuit of high-performance epidermal electronic devices that can efficiently collect and process a diverse range of biological signals is of paramount importance in this field [11].

**Figure 1:**
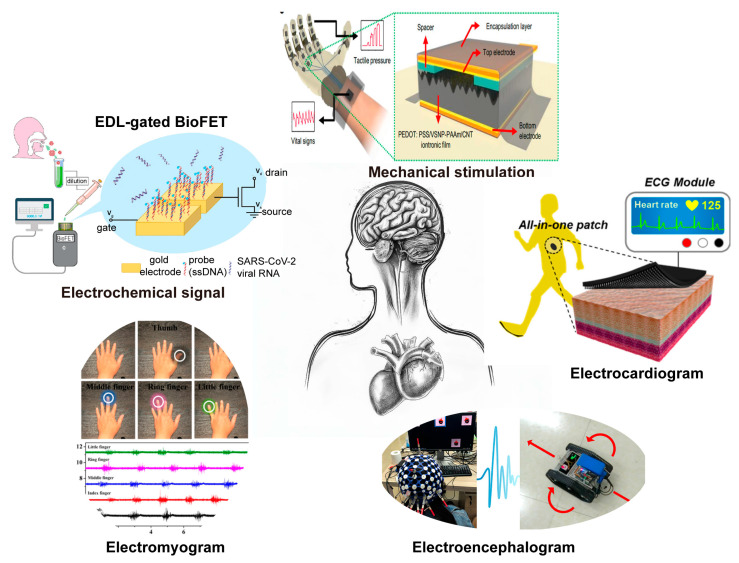
Representative applications of epidermal electronics in healthcare and human-machine interaction [24,25,26,27,28]. Reproduced with permission from [24,25,26,27,28].

Epidermal devices show great potential in health monitoring due to their inherent advantages in flexibility, biocompatibility, and permeability. These properties enable them to seamlessly conform to the skin’s surface, reducing discomfort and interference with normal skin function. Moreover, their ability to continuously monitor various physiological signals in real-time meets the escalating demand for rapid diagnostics and treatment in modern healthcare. This real-time and continuous monitoring capability of epidermal devices can potentially revolutionize healthcare by enabling early detection. A promising solution to the development of high-performance epidermal electronics involves the design and fabrication of devices based on the electrical double layer (EDL) effect [12,13]. EDLs, typically formed at the interface between electrolytes and conductors, offer substantial capacitance due to the EDL effect. In epidermal electronics, EDLs can be harnessed as tactile sensors by exploiting the capacitive characteristics contributed by the EDL capacitance formed at electrolyte-conductor interfaces [14]. These sensors can respond to both static and dynamic stimuli. Additionally, EDL-based devices can serve as biosensors to extract physiological signals with a high signal-to-noise ratio, such as electroencephalogram (EEG) and electromyography (EMG), leveraging the EDL effect. The performance of EDL-based epidermal electronic devices can be further enhanced by optimizing the materials, structures, and system designs. Furthermore, EDL-based devices can respond to mechanical stimulation, making them suitable for applications that require the detection of physical changes or movements. This feature, combined with their ability to process electrochemical signals, broadens their potential use in health monitoring and diagnostics. These properties of EDL are illustrated in Figure 1.

This paper aims to provide a comprehensive review of the current status of EDL-based epidermal electronics, covering material synthesis, device fabrication, integration technology, and device applications. We will discuss the various materials employed in the construction of epidermal electronic devices [15,16,17,18], including metallic materials, polymeric materials, and biomaterials. The influence of these materials’ mechanical and electrical properties, as well as their biocompatibility, will be emphasized [19,20,21].

We will also delve into device fabrication and integration technology, offering insights into the methods for fabricating flexible devices and systems capable of adhering to the skin. Furthermore, we will present the potential applications of EDL-based devices in mechanical signal sensing [22], bioelectrical signal monitoring [21], and electrochemical sensing [23]. It is our hope that this review will serve as a comprehensive guide to the current state and future prospects of epidermal electronics, laying a solid foundation for the development of the next generation of wearable electronics.

## 2. Material Synthesis

In the field of electrochemistry, the Electric Double Layer (EDL) stands out as a molecularly thin interface separating two substances populated by electrically charged particles. These can include an electrode from a solid material and an electrolyte solution [29]. Constituted of two layers of opposite charges, the EDL is structured by the adsorption of ions on the solid surface, thereby forming one layer. Concurrently, the other layer forms within the proximate solution near the surface. Owing to the separation between these charges, the EDL possesses a remarkably high capacitance. Consequently, the EDL holds a pivotal role in affecting various physical and chemical processes unfolding at the interface.

Owning to the advantages of EDL, an EDL electrode emerges as an electrode to store electric charge at the electrolyte solution interface [30]. By their high charge density and rapid charge-discharge rates, EDLs are attractive in applications, such as electrochemical capacitors, sensors, and actuators. However, it is imperative to note that the performance relies on the factors, such as electrode material and electrolyte composition [31] as presented in Table 1.

Also, an Electric Double Layer Transistor (EDLT) can be designed and fabricated based on the EDL effect [32]. This unique type of transistor employs an electrolyte solution as the gate dielectric. By inducing a significant volume of charge carriers through the EDL, the EDLT can modulate the electrical transport in the transistor channel. With low voltage operation, high transconductance, and compatibility with organic semiconductors, EDLTs extend their potential use into the production of flexible, stretchable, and biocompatible electronic circuits. EDLT can be applied in the fields of biosensors, neuromorphic devices, and memory devices [33].

**Table 1:** The representative materials for the flexible and epidermal electronics.

Material	Electrical Conductivity (S/m)	Mechanical Strength (MPa)	Reference
Gold	4.1 × 10^7^	120	[34]
Silver	6.3 × 10^7^	250	[34]
Copper	5.8 × 10^6^	210	[34]
Polyimide	1 × 10^−14^	50–170	[35]
PDMS	8130	1–5	[35]
PET	1 × 10^−14^	50–80	[35]
Polyaniline	5	20–100	[36]
Polypyrrole	40–200	30–80	[36]
PEDOT	300–1000	10–20	[36]
Galinstan	3.46 × 10^6^	N/A	[37]
Silver Nanowire	1 × 10^4^–8 × 10^5^	10–100	[38]
Collagen	10^−6^–10^−4^	0.1–1	[39]
Chitosan	10^−5^–10^−4^	1–5	[39]
Hyaluronic Acid	~10^−3^	0.05–0.5	[39]
PCL (Poly-caprolactone)	2.1 × 10^−14^	5–50	[40]
PLA (Polylactic acid)	10^−15^–10^−13^	40–60	[40]

### 2.1. Metal Based Materials

Galinstan [41] and silver nanowires (AgNW) [42] are typical representatives of metal-based materials and have emerged as prominent options offering flexibility [43], transparency [44], and biocompatibility for the devices. These materials present innovative solutions for biomedicine, wearable devices, and other related applications. In addition, a number of researches have been carried out to further enhance the material properties, such as the development of self-healing [45], shape-memory [46], and stimuli-responsive materials [47]. Also, researchers investigated hybrid materials based on metallic, polymeric, and organic materials to create multifunctional devices with superior performance.

**Figure 2:**
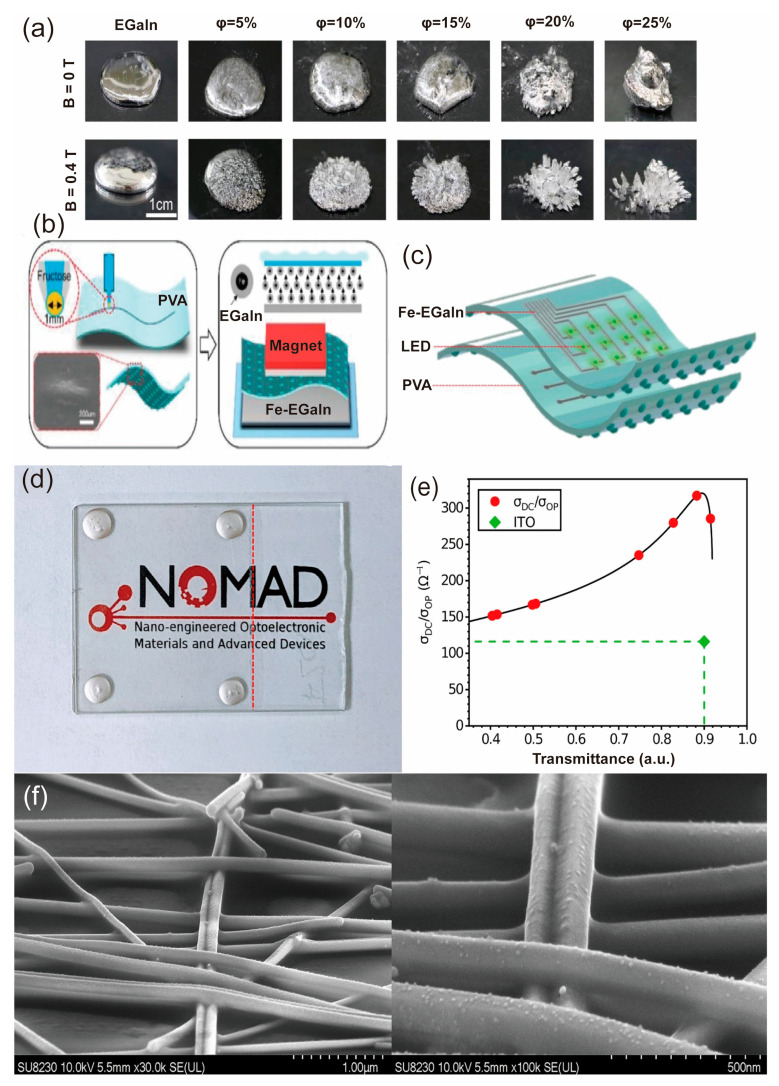
Demonstration of the application of metal-based materials in epidermal electronics [48,53]. (**a**) The varying packing ratios of Fe-EGaIn significantly impact their electrical conductivity. Reproduced with permission from [48]. (**b**) Fabrication process of Fe-EGaIn-based device [48]. (**c**) the schematic diagram for the device. Reproduced with permission from [48]. (**d**) Display of light transmission qualities in silver nanowire-based transparent conductive films Reproduced with permission from [53]. (**e**) An illustration of the superior transparency characteristics of silver nanowires. Reproduced with permission from [53]. (**f**) The SEM image of the Ag nanowire. Reproduced with permission from [53].

The use of liquid metals, such as Magnetic Liquid Metal (Fe-EGaIn) and Galinstan, enables exceptional stretchability and electrical conductivity of the devices, which are able to overcome the limitations of traditional solid metals. In 2019, Guo et al. explored the potential application of Fe-EGaIn-based liquid metals in multifunctional electronics [48]. As shown in Figure 2a, Fe-EGaIn has unique properties in remote magnetic self-healing, water degradation, and thermal transfer printing. Figure 2b,c demonstrate the fabrication process for Fe-EGaIn-based devices and their application in LED circuits. It is possible that this discovery possesses significant potential for various applications in the fields of stretchable electronics, recyclable devices, and soft robots.

Nanowires, as another representative of metal-based materials, have also shown great potential in the development of flexible, transparent, and biocompatible devices due to their excellent electrical and mechanical properties [49]. Among the various nanowires, AgNW is a notable example of nanowires that possess excellent electrical and mechanical properties while maintaining flexibility, transparency, and biocompatibility [50]. Therefore, it has been widely employed in the fabrication of transparent conductive films for flexible and wearable electronics [51,52]. Gerlein et al. demonstrated AgNW-based transparent conductive films for flexible and wearable electronics [53]. Figure 2d,e shows the AgNW-based device with high electrical conductivity, excellent mechanical flexibility, and optical transparency. Figure 2f shows the microstructure of AgNW. These advantages facilitated the development of highly conformable and stretchable electronic devices, enhancing their adaptability to various applications.

The advancements in metal-based materials, especially liquid metals and nanowires, have significantly contributed to the progress of epidermal electronic devices. These materials offer unique properties that enable the fabrication of flexible, transparent, and biocompatible devices suitable for a wide range of applications, including biomedicine, wearable electronics, and human-machine interaction. Further research and development in this field is expected based on the advanced materials and device structure for the improvement of performance and functionality.

### 2.2. Polymer-Based Materials

Recently, polymeric materials, such as polyimide (PI) [54], polydimethylsiloxane (PDMS) [55], and polyethylene terephthalate (PET) [56], which provide lightweight, highly flexible, and processable alternative properties, were also employed to be used as substrates and functional layers.

**Figure 3:**
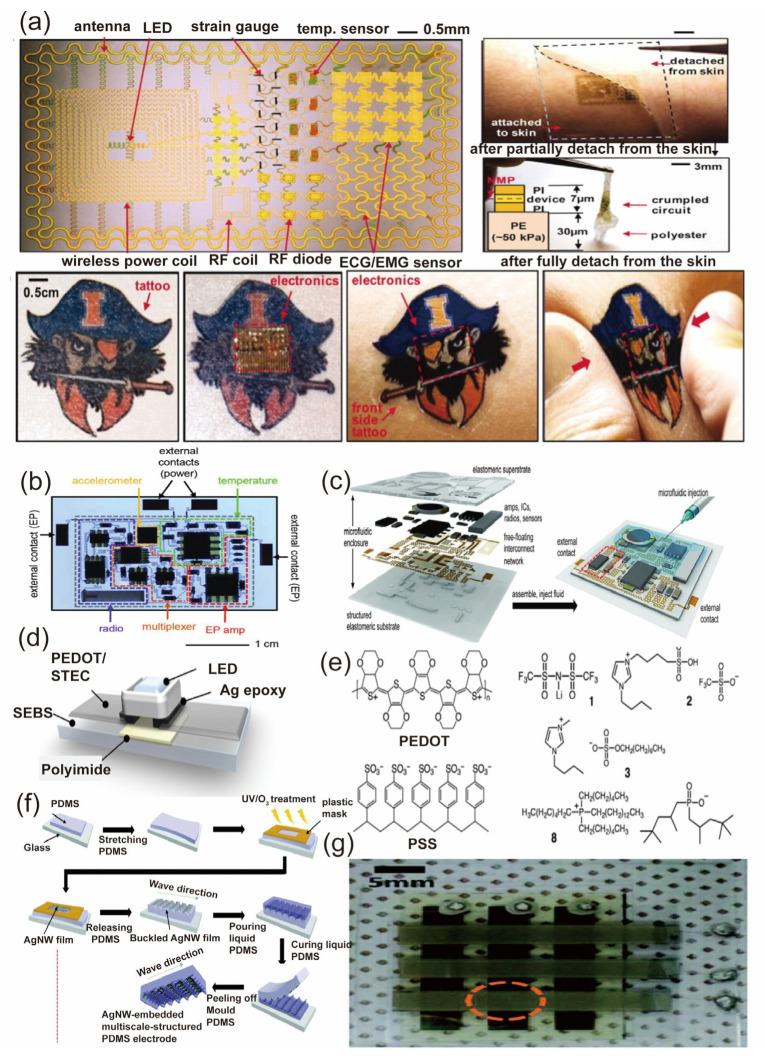
Presentation of Polymer-Based Materials [57,58,62,63]. (**a**) Schematic diagram for a flexible system with sensors, stimulators, energy harvesting systems, and logic components on polymide substrate. Reproduced with permission from [57] (**b**) The optical image displays colored dashed boxes with labels, identifying the different subsystems of the multifunctional wireless sensor. Additionally, a gray dashed box surrounding the periphery indicates the approximate location of bonding the superstrate. Along with the image, operational data from the sensor is also available. Reproduced with permission from [58] (**c**) The exploded-view schematic illustration showcases the essential components of the soft, stretchable electronic system. Furthermore, there are photographs depicting the system after assembly, highlighting the initial stages of microfluidic injection with a syringe, all within a thin elastomeric microfluidic enclosure. Reproduced with permission from [58] (**d**) Schematic representation of an LED device, where PEDOT wires bridge the device to the power source. Reproduced with permission from [62]. (**e**) Chemical structure for the PEDOT and PSS. Reproduced with permission from [62]. (**f**) Schematic diagrams depicting the fabrication process of the multiscale-structured PDMS electrode embedded with AgNWs, as well as the capacitive pressure sensor. Reproduced with permission from [63]. (**g**) A photograph showcasing a sensor array with a 3 × 3 configuration of pixel-type pressure sensors. Reproduced with permission from [63].

In 2011, Kim et al. demonstrated the feasibility of using polyimide as a substrate for the fabrication of stretchable and flexible electronic devices [57]. They successfully fabricated a transparent and stretchable conductor using a combination of graphene and a polyimide substrate. The device exhibited excellent mechanical and electrical properties, highlighting the potential of polymeric materials in the application of flexible electronics, as shown in Figure 3a. Additionally, it has been made possible to use nanotechnology to create flexible, multifunctional epidermal devices that include sensors, stimulators, energy harvesting systems, and logic components. A flexible and stretchable electrical device with PDMS was created by Xu et al. in 2013 for use in human-machine interactions and health monitoring [58]. This device is depicted in Figure 3b.

Their innovation in integrating thin-film transistors, temperature sensors, and strain gauges on a polyimide substrate shows the potential of the fabrication of micro and nanodevices with polymeric materials. With the employment of polymeric materials and various fabrication technologies, researchers enhanced device performance by optimizing the properties and functionality of these materials, which led to a substantial leap in the functionality and versatility of the devices. The essential components of the soft, stretchable electronic system are vividly displayed in Figure 3c. In addition, the system, post-assembly, is captured in detail through photographs. These visuals underscore the preliminary phases of the microfluidic injection process, carried out with a syringe, all contained within a sleek, elastomeric microfluidic enclosure.

While polymeric materials have been widely employed in flexible electronics, their low electrical conductivity poses a challenge to further enhance the device’s performance. To overcome this issue, conductive polymers such as polyaniline [59], polypyrrole [60], and poly(3,4-ethylenedioxythiophene) (PEDOT) [61] were developed. In addition, through doping these conductive polymers with other materials like carbon nanotubes, graphene, and metallic nanoparticles, researchers were able to further enhance their electrical conductivity. In 2015, a study by Wang et al. showed the potential of PEDOT:PSS in fabricating flexible [62], transparent, and highly conductive electrodes, as shown in Figure 3d,e. It is found that the hybrid material displayed superior electrical conductivity and mechanical stability compared to its individual counterparts. In 2015, Joo et al. reported a highly stretchable, self-healing, and conductive nanocomposite material by incorporating silver nanoparticles into a PDMS matrix and the fabrication process of the device with the materials as shown in Figure 3f [63]. The devices showed promising applications in wearable electronics, sensors, and actuators due to their excellent mechanical and electrical properties, as shown in Figure 3g. In addition, they demonstrated that the nanocomposite material could be used to fabricate a self-healing strain sensor that could recover its functionality after being damaged by cutting or tearing.

To better understand the working mechanism of the EDL effect in these materials, it is important to develop theoretical and experimental methods that can capture the microscopic structure of the electrochemical interface and its working mechanisms. However, modeling and measuring the EDL structure for complex systems involving conductive polymers and their hybrid materials remains a challenge. For example, the effects of polymer chain conformation, molecular interactions, and ion solvation on EDL behavior are not fully understood. Therefore, further research is needed to explore the EDL effect in these materials and reveal their implications for flexible and wearable electronics [64,65,66].

### 2.3. Bioinspired-Materials

**Figure 4:**
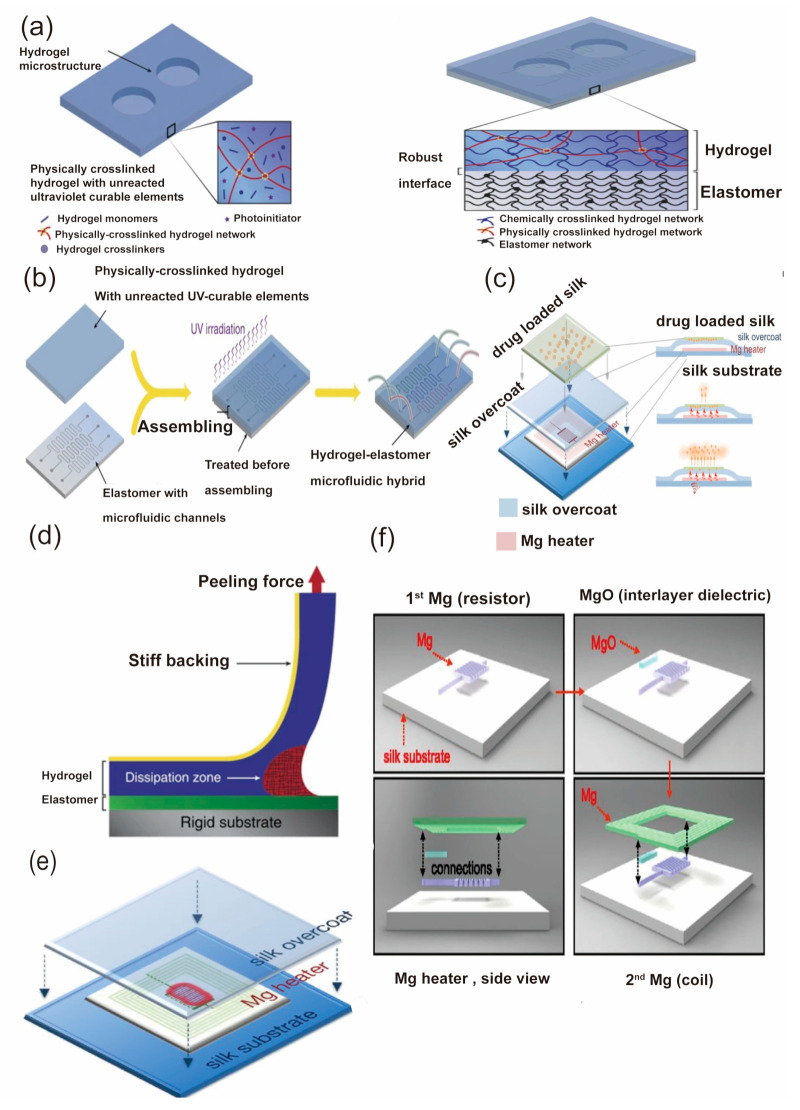
Representatives of bioinspired materials [72,75] (**a**) The hydrogel-elastomer hybrid forms extremely robust interfaces after ultraviolet irradiation due to the covalently anchored polymer network in the hydrogel on the elastomer surface. Reproduced with permission from [72]. (**b**) The image depicts a schematic representation of the fabrication process involved in creating a hydrogel-elastomer microfluidic chip. Reproduced with permission from [72]. (**c**) Schematic diagram of a drug delivery device based on silk material. Reproduced with permission from [75]. (**d**) Schematic diagram of the peeling force measurement of the biomaterial-based materials. Reproduced with permission from [72]. (**e**,**f**): The image presents the device fabrication and RF-thermal response characterization. (**e**) The packaging of the magnesium heater in a protective silk pocket. (**f**) The fabrication process of a fully dissolvable wireless heating device. Reproduced with permission from [75].

Biomaterials have become more attractive for the application of epidermal electronics due to their high biocompatibility and degradability. Researchers have employed biomaterials to develop highly biocompatible, biodegradable flexible sensors, stimulators, drug delivery devices, and artificial neural networks [57,66,67]. Materials like silk fibroin, chitosan, and cellulose, which show potential for long-term stability and conformability with the human body [68,69,70], have been extensively investigated for their application in flexible devices. Also, hydrogel, a highly hydrated network of polymers, has been employed to develop sensors and actuators since it is able to closely mimic the properties of biological tissues [71]. For example, Yuk et al. developed a hydrogel-based electronic skin (e-skin) as shown in Figure 4a, and an improvement in the performance was obtained after UV irradiation [72]. As shown in Figure 4a, the hybrid material composed of Alg-PA hydrogel and PDMS elastomer exhibits a highly robust interface after exposure to UV irradiation with a wavelength of 365 nm, and the original microstructure of the material, such as microfluidic channels or electrical circuits, remains intact in the hybrid [72]. Figure 4b illustrates the fabrication procedure for this device, which shows self-healing properties and is able to sense pressure and temperature signals simultaneously. Figure 4c,d demonstrates the adhesion properties of the materials and the fabrication process of the drug delivery device based on silk material. This result shows the potential application of hydrogel in biocompatible and multifunctional devices. Moreover, silk fibroin has also emerged as a promising biomaterial for the development of flexible electronics due to its biocompatibility, biodegradability, and mechanical strength [73,74]. Tao et al. demonstrated the fabrication of a silk fibroin-based flexible pressure sensor [75], which shows potential in wearable electronics, healthcare monitoring, and human-machine interfaces, as shown in Figure 4e,f. Correspondingly, Aziz et al. developed a highly conductive and amorphous biopolymer blend electrolyte based on chitosan and dextran for EDL applications [76].

The exploration of EDL phenomena in various material systems has significantly contributed to the development of next-generation electronic devices, especially for epidermal electronics. These advancements in material synthesis have been addressing some challenges in this field, such as the long-term stability of devices, signal processing and data transmission efficiency, and improving wearing comfort [77,78]. Studying metal-based materials like Galinstan and AgNWs has opened up new possibili ties, especially in biomedicine and wearable devices. Polymeric materials and biomaterials, including Polyimide, PDMS, and PET, are also gaining attention in the development of substrates and functional layers due to their lightweight, flexible, and biocompatible properties. However, enhancing their electrical conductivity remains a challenge [79,80,81]. Bio-inspired materials with high biocompatibility and degradability are particularly appealing for long-term wearable devices. Nevertheless, there is still a need for more research and innovation to address concerns about long-term stability, signal processing optimization, and user comfort. Further research in material synthesis is crucial to advancing the scalability and cost-effective manufacturing of these technologies [82].

Table 2 summarizes the designs of electric double layer (EDL) materials used in the fields of diagnostics and drug delivery. These designs utilize various types of materials, such as metals, metal oxides, polymers, and composites, and their desired outcomes span a range of medical applications.

## 3. Device Fabrication

Device fabrication is also a key factor in determining the performance of epidermal electronics. Additive manufacturing and subtractive manufacturing are the two primary techniques used to fabricate complex and multi-functional devices. In this section, we examine the representative techniques in relation to the fabrication of epidermal electronic devices and explore the integration strategies for flexible and rigid components.

### 3.1. Subtractive Manufacturing

Subtractive manufacturing enables precise patterning of the materials and is considered a crucial technique in the fabrication of epidermal electronic devices. As researchers strive to optimize the fabrication process, it is essential to consider the unique requirements of each device. Factors such as resolution, throughput, and processing compatibility are usually taken into consideration. While each technique has its advantages and drawbacks, advancements in subtractive manufacturing, along with the development of novel hybrid and additive processes, will contribute to the development of epidermal electronic devices by enhancing their performance, functionality, and conformability with the human body.

**Figure 5:**
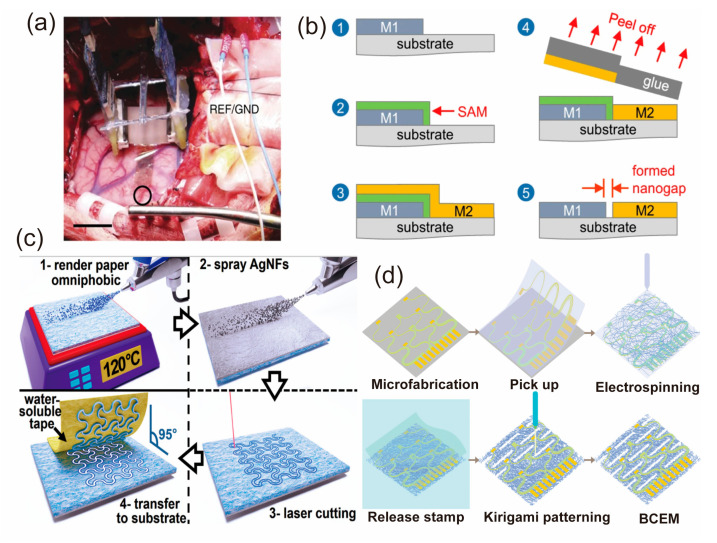
Schematic diagram for typical subtractive manufacturing methods [87,88,89,90]. (**a**) The NeuroGrid is designed to conform to the surface of the rat somatosensory cortex. In the image, the edge of the resected dura can be seen at the top left of the craniotomy, with a scale bar indicating a measurement of 1 mm. Reproduced with permission from [87]. (**b**) The development of metal nanogaps through adhesion lithography. Reproduced with permission from [88]. (**c**) The fabrication of wearable and implantable epidermal paper-based electronic devices based on a paper-based laser cutting method. Reproduced with permission from [89]. (**d**) Transfer-based laser cutting for the fabrication of electrophysiological signal sensor. Reproduced with permission from [90].

**Lithography** is a versatile manufacturing technique that utilizes light to pattern the thin film. As a result, a desired shape or structure can be defined. This technique is widely used in epidermal electronics, where lithography can be applied to produce thin, flexible, and conformable devices with microstructures to monitor various physiological signals. In a study by Khodagholy et al. [87], researchers utilized lithography to fabricate high-density conformable electrode arrays for epidermal electronic applications, as shown in Figure 5a. This device can record local field potentials (LFPs) and neuronal action potentials (APs) from the brain surface without penetrating the brain surface. This neural interface array is based on organic materials and is ultra-compliant, biocompatible, and scalable, allowing coverage of large cortical areas while maintaining high resolution and stability. In 2018, Semple et al. reported a lithography method based on a single self-assembled monolayer (SAM), as shown in Figure 5b [88]. This technique enables the preparation of electrodes with a resolution of 15 nm and has been successfully applied to a wide range of flexible electronic devices such as high-speed Schottky diodes, memory resistors, highly sensitive photodiodes, metal oxide transistor arrays, RF diodes, and nano-LEDs.

**Laser cutting** is a technique that uses a high-power laser beam to cut materials into desired patterns, with advantages in fast speed, low cost, and high accuracy. This method can also be applied to fabricate epidermal electronics that can conform to the skin and have sensing, heating, and communication functions. For example, Sadri et al. developed paper-based epidermal electronic devices for wearable and implantable applications by using spray-based deposition of salinizing agents and conductive inks followed by laser cutting technology to create paper-based devices with different shapes and functions [89]. The detailed fabrication process is illustrated in Figure 5c. Li et al. reported a stamp transfer-based method that enables hybrid integration of high performance micromachined electronics with a porous, flexible substrate of electrospun fibers [90]. The laser cutting technology was used to realize the Kirigmai structures of the device [63]. The fabricated device shows excellent permeability and conformability and can be used for the recording of high quality electrophysiological signals, as shown in Figure 5d.

These subtractive manufacturing techniques provide promising solutions for developing epidermal electronics with high resolution. They also pave the way for on-demand fabrication and customization of epidermal electronics for diverse applications. However, the waste of the material, the requirement of expensive equipment, and the process combability are still challenges for the fabrication of flexible and stretchable electronics.

### 3.2. Additive Manufacturing

**Inkjet printing** is another method for the fabrication of epidermal electronics, enabling the precise deposition of conductive, semi-conductive, or insulating materials onto a flexible substrate. This technique uses inkjet printer nozzles to accurately eject ink droplets, forming a patterned circuit on the substrate. Despite its high resolution, cost-effectiveness, and scalability, inkjet printing faces challenges such as ink dispersion and film formation, which can hinder the creation of uniform and well-controlled layers.

**Figure 6:**
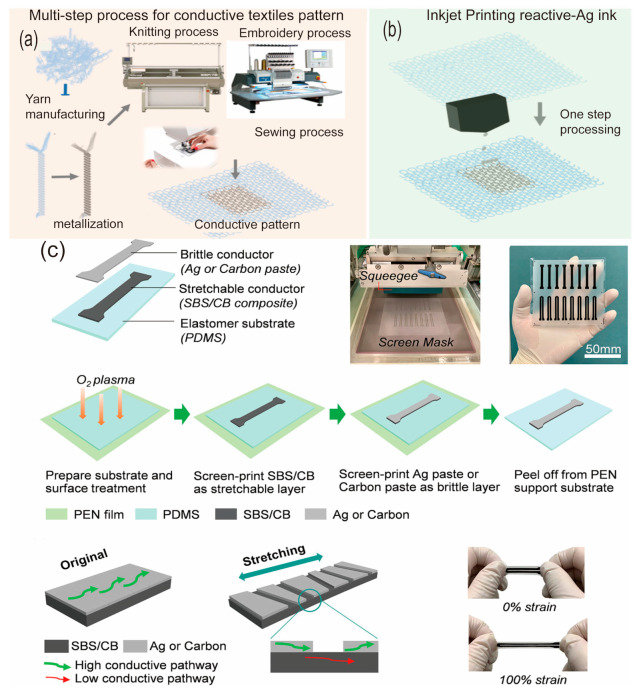
Schematic diagram for typical additive manufacturing methods [91,92]. (**a**) The schematic diagram for conductive textile patterns. Reproduced with permission from [91]. (**b**) The patterning of conductive structures on textiles using the inkjet printing method. Reproduced with permission from [91]. (**c**) The fabrication of strain sensors based on the screen-printing method. Reproduced with permission from [92].

Shahariar et al. explored the potential of inkjet printing on textiles for the integration of passive electronic devices and sensors [91]. They proposed a novel inkjet printing process that uses particle-free reactive silver ink on uncoated polyester textile knit, woven, and nonwoven fabrics. This method allows the ink to conformally coat individual fibers, creating a conductive network within the textile structure without altering its durability and mechanical behavior. They also found that the electrical conductivity of the inkjet-printed conductive coating could be significantly improved through in situ heat-curing during the printing process. Figure 6a,b demonstrate the raw materials and the fabrication process. This review summarizes the potential of inkjet printing in epidermal electronics and offers a low-cost, scalable, and automated manufacturing process.

**Screen printing** This technique is able to transfer material with a designed pattern onto a stretchable and flexible substrate using a stencil or mesh screen. In brief, the substrate is covered with the screen, which is then used to squeegee the ink through the mesh openings and onto the substrate, a circuit with the desired pattern can be realized. For example, Wang et al. demonstrated the potential of screen printing for fabricating stretchable and wearable strain sensors [92]. However, their resolution is limited compared to other additive methods, making them less suitable for highly miniaturized devices or those requiring extremely fine features. Screen printing is a simple and low-cost technique for integrating sensors into fabrics or other substrates that can be worn on the skin. Wang et al. highlighted the integration of strain sensors into fabrics through screen printing [92]. This integration allows the creation of garments capable of monitoring body movement, posture, and vital signs. These advancements have made a significant contribution to sports, fitness, and medical applications, enabling real-time tracking and analysis of physical parameters. The related progress of screen printing and its applications is summarized in Figure 6c.

**Figure 7:**
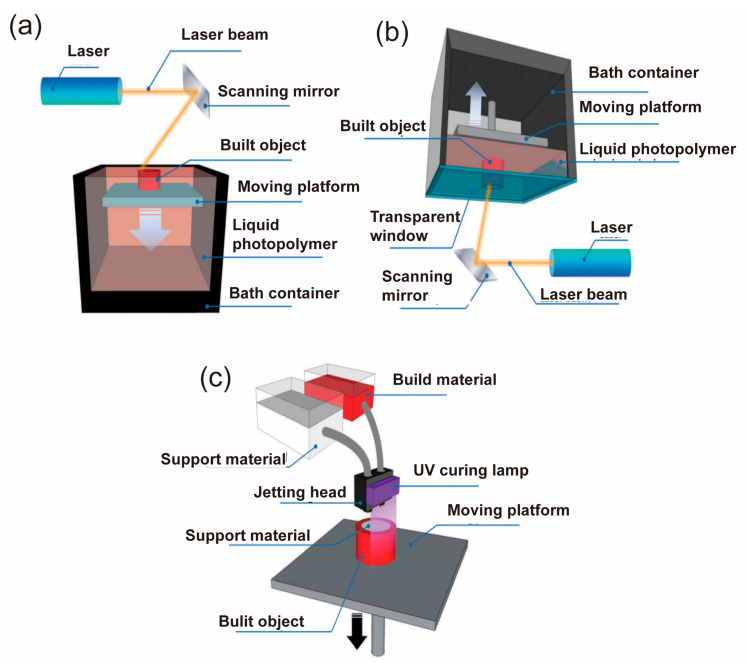
The Schematic diagram for three-dimensional (3D) printing [96]. (**a**) Stereolithography (SLA) in a bath configuration initiates polymerization by scanning a laser across liquid resin. This process continues in layers, with a descending table exposing fresh resin. Reproduced with permission from [96]. (**b**) The layer configuration of SLA directs a laser through a tank’s transparent bottom, adhering the polymerized layer to the table. The table moves upwards to create a gap refilled with fresh resin for the next layer. Reproduced with permission from [96]. (**c**) Material jetting involves ink-jetting photocurable material onto a tray with supporting material. A UV light hardens each layer. Following this, the table moves downwards and the process is repeated with new material until the desired object is formed. Reproduced with permission from [96].

**Three-dimensional (3D) printing** This method can be used to fabricate three-dimensional devices layer by layer, enabling the fabrication of biocompatible electrodes and wearable electronics with complex structures, as shown in Figure 7. One of the 3D printing processes is Stereolithography (SLA), which converts liquid resin into solid layers by curing it with a laser, creating models, prototypes, patterns, and production parts [93]. Other 3D printing processes include fused deposition molding (FDM), and selective laser sintering (SLS), which have been used for epidermal electronics [94]. However, 3D printing is a relatively low efficiency and low resolution additive manufacturing technique. Recently, advances in multi-material and high-resolution printing have been investigated to overcome these limitations [95,96]. 3D printing is a promising technique for creating customized flexible devices that can conform to the skin’s surface and adapt to its deformation.

**Figure 8:**
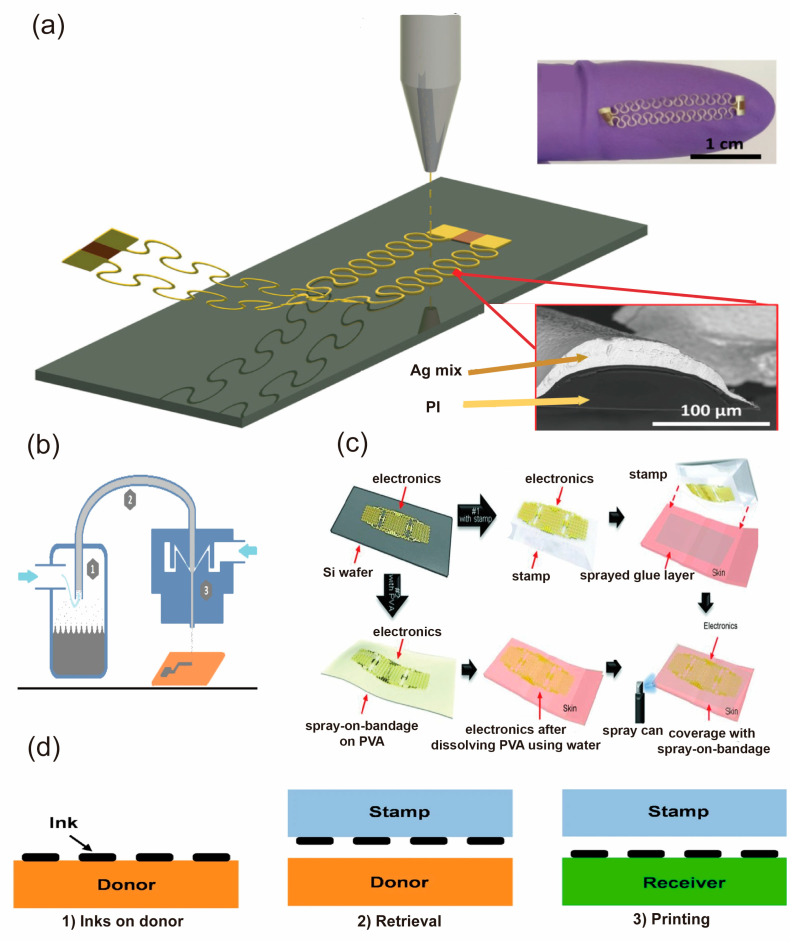
Demonstration of transfer printing and aerosol jet printing [97,99,100,102]. (**a**) The fabrication process of freestanding stretchable conductive wires using aerosol-jet printing. Reproduced with permission from [97]. (**b**) The process of ink atomization. Reproduced with permission from [99]. (**c**) The fabrication of physiological sensors by the transfer printing method. Reproduced with permission from [100]. (**d**) The process for transfer printing. Reproduced with permission from [102].

**Aerosol jet printing** is an innovative technique that employs a carrier gas to deposit a fine mist of material onto a substrate, which is then cured using a laser or other methods. This method has the advantages of high resolution, low-temperature processing, and direct writing on various substrates, and it is widely employed in the fabrication of epidermal electronics. Jing et al. demonstrated a novel approach to the fabrication of freestanding functional structures layer by layer with aerosol jet printing [97]. These structures can serve as stretchable interconnects or electrodes, strain sensors, and humidity sensors in wearable systems, as shown in Figure 8a. In 2021, Mchela et al. provided a method that employs Aerosol jet printing that can fabricate a sensor that can be used to make energy storage electrodes and sensing elements based on Ti3C2Tx printed by this method on flexible substrates and explores the temperature influence on the resistance of the device [98]. The study reveals that there is an increase in resistance with temperature due to the thermoelectric effect and the thermal expansion mismatch between the ink and substrate. Skarżyński et al. employed aerosol jet printing techniques to fabricate degradable epidermal electronics [99], which used silver nanopowder, toluene, surfactant, and polyimide film. Silver nanopowder is the functional phase and is used to form the conductive path. Toluene is the solvent and is used to adjust the viscosity and surface tension of the ink. Surfactants are additives used to improve the ink’s atomization efficiency, print line uniformity, and resistivity. Polyimide film is the substrate, used to carry the print line and provide flexibility and stability, demonstrating the potential for these devices to degrade in vivo and minimize their environmental impact, as shown in Figure 8b. This fabrication process can also be used for interconnects and passive and active electronic components, such as top-gated field-effect thin-film transistors. These results indicate that this fabrication process can be used for flexible, wearable, biocompatible sensors, and electronics.

**Transfer printing** is a sophisticated technology that integrates additive and subtractive manufacturing techniques to enable the precise and efficient fabrication of flexible devices [100], such as stretchable strain sensors [101], radio frequency (RF) power harvesters [102], and transistors [103].

The process initiates the fabrication of a temporary substrate with specific patterns using the substrate method. Then, functional materials, such as conductive inks or nanomaterials, are deposited on the patterned substrate using an additive technique. Afterwards, the materials are transferred from the temporary substrate to a flexible or stretchable target substrate using methods like micro-contact printing or hot-press bonding. This technology provides a solution for the fabrication of devices on an arbitrary substrate that is able to seamlessly adhere to the surface of the human body. In 2013, Yeo et al. reported a novel method for printing electronics and sensors directly onto the skin [100]. Printed electronics measure a variety of physiological signals relevant to health and wellness, such as ECG, EMG, skin temperature, and skin hydration, as well as their biocompatibility, durability, and wireless communication capabilities. Multifunctional epidermal electronic devices are printed directly onto the skin surface by solvent engineering techniques. The multifunctional device is able to record various physiological signals related to health and wellness, as shown in Figure 8c. Transfer printing technology for flexible and stretchable inorganic electronics is outlined through a discussion of future developments and applications as reported by Linghu et al. [102]. They stress the process of transfer printing, as shown in Figure 8d.

In the field of wearable technology, additive manufacturing has emerged as an important technique for customizable epidermal electronic devices that adapt to the user’s unique skin characteristics and requirements. In addition, additive manufacturing significantly reduces material waste and environmental impact by reducing pollution, increasing production efficiency, and promoting sustainable practices in the development of portable, flexible electronic devices. Also, this fabrication technology is able to improve the biodegradability of the devices by incorporating environmentally friendly biodegradable polymers. As a result, the end-of-life disposal of these devices results in minimal environmental impact, as they undergo a decomposition process that breaks down the constituent materials into the environment.

Significant progress in fabrication techniques for epidermal electronics has been achieved in recent years. In terms of subtractive manufacturing, the application of lithography and laser cutting techniques has been demonstrated for patterning and creating microstructures of epidermal electronic devices [87,88]. These processes provide high-resolution, functional, and conformable devices, which are critical for the optimization of biocompatible interfaces with the human body. However, these methods have significant challenges, such as high costs, process compatibility, and material waste. Additive manufacturing approaches could supplement these drawbacks, opening a pathway for continuous improvement in epidermal electronic device development. Advancements in additive manufacturing techniques, such as inkjet printing, screen printing, 3D printing, aerosol jet printing, and transfer printing exhibit promising developments for epidermal electronics. Each of these techniques presents unique benefits and potential challenges and incorporating additive and subtractive manufacturing would be a possible solution to overcome the challenges [91]. Furthermore, fabrication techniques will also need to consider issues related to the biocompatibility, environmental impact, and degradation of devices. For instance, the advent of biodegradable epidermal electronics through aerosol jet printing represents an important direction for reducing the environmental footprint of these devices [99]. Future research should aim to enhance the durability and biodegradability of these devices while ensuring their effective functionality.

Table 3 provides a comparison of various fabrication methods, including lithography, laser cutting, inkjet printing, screen printing, 3D printing, aerosol jet printing, and transfer printing. For each method, the table identifies suitable materials, the resolution that can be achieved, the speed at which fabrication occurs, and relevant references for further exploration.

## 4. Integration Techniques for Rigid and Flexible Components

To achieve the desired functionality, comfort, and reliability of epidermal electronic devices, rigid and flexible components need to be integrated with high stability. The challenges are related to mechanical deformation, electrical connectivity, and thermal management, which are to be discussed in this section, along with various integration techniques used for the connection of rigid and flexible components.

### 4.1. Interconnects for the 2D and 3D Dimensions 

**Figure 9:**
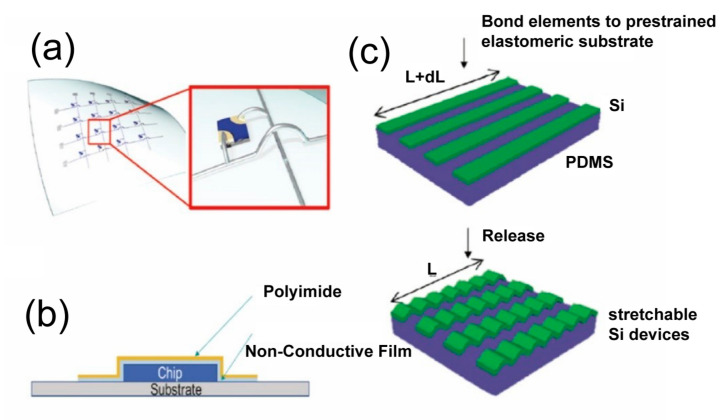
Representative integration techniques for rigid and flexible components [107,109,111]. (**a**) Interconnects in 2D and 3D dimensions for device integration. Reproduced with permission from [107]. (**b**) Epoxy-based bonding. Reproduced with permission from [109]. (**c**) ACF bonding. Reproduced with permission from [111].

Interconnects serve as a critical component within the architecture of epidermal electronic devices, facilitating essential electrical connections among various components [104,105,106]. Numerous strategies exist for the realization of 2D and 3D interconnects, with printing emerging as a prevalent method [107]. To accommodate strain and bending, these interconnections can adopt an array of geometries, such as serpentine, helical, or buckled shapes, as depicted in Figure 9a. These geometric configurations ensure the preservation of electrical connectivity and mechanical integrity under deformation, thereby guaranteeing the seamless operation of the epidermal electronic devices during routine activities.

### 4.2. Rigid IC Chip Bonding to Soft Substrates 

This procedure entails the utilization of adhesives, solder, or other bonding agents to secure rigid chips onto flexible substrates [108,109,110]. The objective is to establish robust electrical contact, mechanical stability, and thermal compatibility. The choice of an appropriate bonding technique is pivotal to the success of this process. A variety of bonding methods are frequently employed, each presenting its own unique advantages and considerations.

### 4.3. Anisotropic Conductive Film (ACF) Bonding 

ACF, a composite material composed of conductive particles and adhesive, serves a dual function: it provides mechanical adhesion and establishes an electrical connection between the rigid chip and the flexible substrate [111,112,113]. The specific application of ACF in the bonding process and the resulting structure are illustrated in Figure 9c.

### 4.4. Epoxy-Based Bonding 

Epoxy adhesives, known for their superior adhesion to a wide range of materials and high mechanical strength, are considered suitable candidates for bonding rigid chips to flexible substrates [109,114]. Depending on the specific requirements of the device, conductive epoxies or different interconnects, such as wire bonding or flip-chip methods, can be employed to achieve the electrical connection. The application of epoxy-based bonding is presented in Figure 9b.

In the development of epidermal electronic devices, the integration of rigid and flexible components also emerges as an essential consideration. The choice of integration technique depends on the distinctive requirements of the device, including its size, complexity, and performance. Substantial advancement has been made in this area to enhance the stability and functionality of epidermal electronics through continued refinement in integration methods. However, there still remain a number of challenges to be overcome, including long-term mechanical stability, electrical integrity, thermal compatibility, and biocompatibility [115,116].

## 5. Applications of Epidermal Electronics in Diagnosis and Therapeutics

### 5.1. Diagnostic Applications of Epidermal Electronics

#### 5.1.1. Mechanical Signal Monitoring

**Figure 10:**
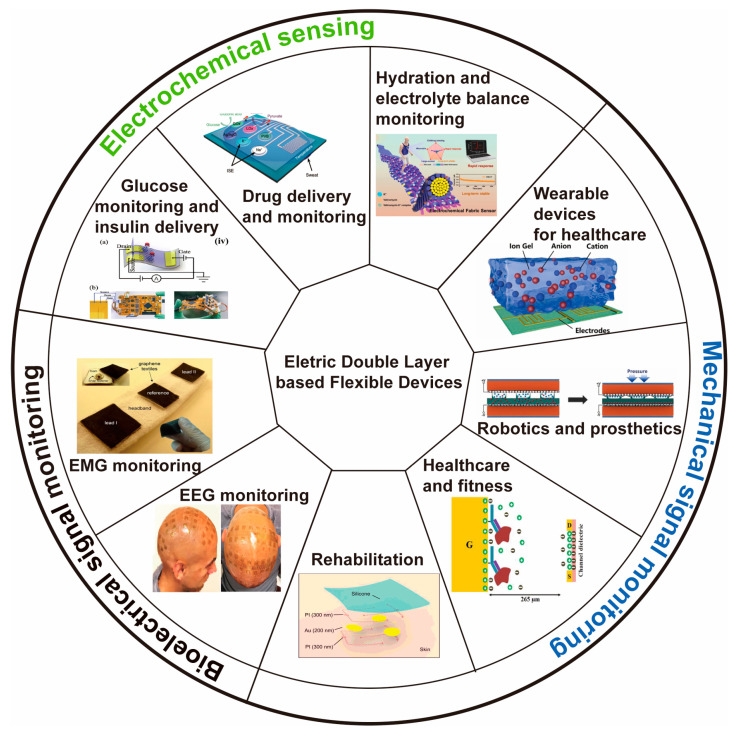
Overview of the representative application of epidermal electronics [117,119,120,121,122,123,124,125,126]. Reproduced with permission from [117,119,120,121,122,123,124,125,126].

Epidermal Dielectric Layer (EDL) capacitive sensors exhibit remarkable sensitivity, a large span, and a short response time, making them an ideal solution for epidermal electronics [117,118]. These sensors have the ability to detect force and human muscle movement and adhere to human skin, thereby providing a versatile platform for various diagnostic applications in healthcare. Figure 10 shows us different applications based on electrically double-layered epidermal electronics.

**Figure 11:**
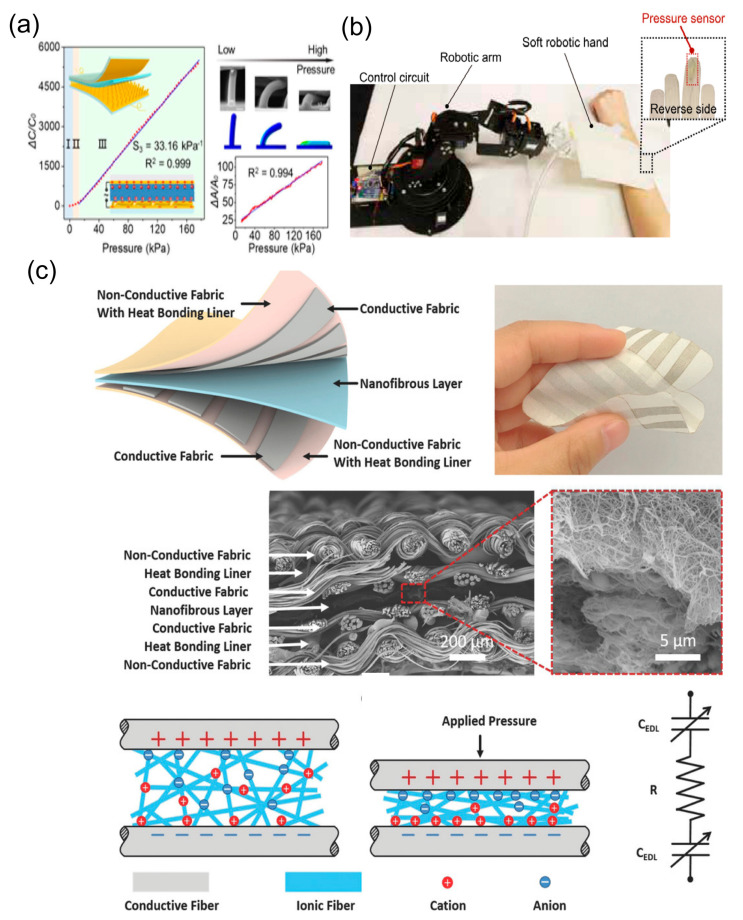
Demonstration of mechanical signal monitoring by flexible sensors [128,129,133]. (**a**) An EDL-based flexible pressure sensor with a nanoscale iontronic interface and high sensitivity and linearity over a wide pressure range was obtained. Reproduced with permission from [128]. (**b**) This image presents the experimental set-up used to demonstrate a sensorized robotic fingertip on a soft hand, which is controlled by a robotic arm. This setup is designed to touch, apply, and measure pressure to and from a human arm. Reproduced with permission from [129]. (**c**) The schematic diagram of the all-fabric sensing matrix featuring a nanofibrous layer, alongside a photograph of the 4 by 4 sensing array with a spatial resolution of 1 by 1 mm. The SEM photos provide cross-sectional views of the device. Reproduced with permission from [133].

EDL pressure sensors prove particularly valuable in prosthetic and robotic limbs, offering precise tactile feedback and control. For instance, Matsuda et al. introduced a highly stretchable sensing array capable of independently detecting pressure and strain [127]. This array was able to detect in-plane biaxial tensile deformation and pressure, thereby facilitating the monitoring of eye blinks, finger movements, and wrist pulses. A similar approach was reported by Lu et al. and they incorporated a nanoscale “iontronic” interface, which is inspired by the operation biological system, with a micropillared electrode. In the context of the reported sensor, the iontronic interface enhanced its performance across a broad sensing range. The sensor demonstrated superior sensitivity and linearity [128], underpinning its potential for applications in health monitoring and intelligent robotics, as depicted in Figure 11a. Su et al. designed another stretchable pressure sensor employing an ionic capacitive sensing mechanism and a hierarchical microstructure. Even under the stress of stretching, the sensor continued to accurately sense pressure [129], indicating its potential use in human and soft robotic skin, as seen in Figure 11b.

In the fields of healthcare and fitness, EDL pressure sensors can deliver real-time feedback on body movement, posture, and muscle activation patterns [130]. This real-time data is helpful in preventing injuries, enhancing athletic performance, and facilitating the development of personalized exercise regimes. In addition, EDL pressure sensors can monitor pulse waves and blood pressure, two pivotal health indicators [77,131,132]. In 2017, Li et al. introduced a flexible supercapacitive sensing modality to fabric materials via an elastic ionic-electronic interface. The novel design offered high sensitivity [133], noise immunity, and remarkable stability for wearable pressure and force sensing, as depicted in Figure 11c. In 2019, Huang et al. developed EDL pressure sensors to detect pulse wave signals and estimate blood pressure non-invasively by attaching the sensor to the wrist or finger [133]. Another compelling application lies in monitoring muscle movements and plantar pressure. In 2022, Hua et al. presented an EDL pressure sensor [134] that could be incorporated into wearable devices like gloves, socks, or shoes. The sensor effectively measured muscle activation and foot pressure distribution during various activities such as walking, running, or cycling, thereby aiding users in improving their posture, balance, and performance.

#### 5.1.2. Bioelectrical Signal Monitoring

Bioelectrical signals hold paramount importance in the monitoring of human physiology and various disease states [135,136,137,138,139]. The EDL-gated FET biosensor array has emerged as an effective platform for probing ion channels and bioelectric signals of circulating tumor cells, signifying a revolutionary advancement in diagnostic procedures.

**Figure 12:**
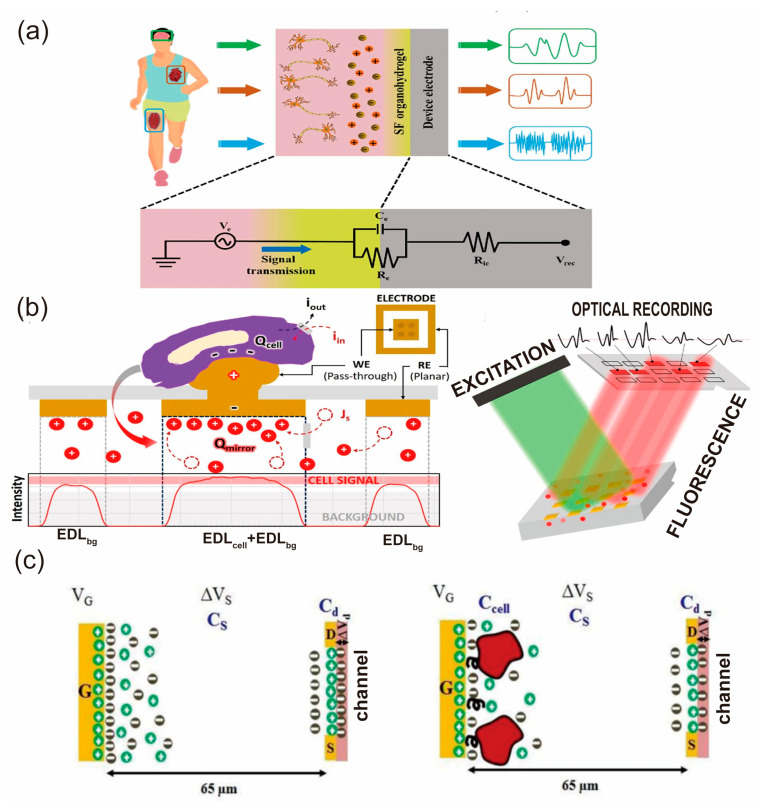
Demonstration of Mechanical signal monitoring by flexible sensors [138,140,141] (**a**) The silk fibroin organohydrogel as an interface between the tissue and electrode for bioelectrical monitoring Reproduced with permission from [140]. (**b**) the working principle of the nanoplatform. The schematic diagram for the device with a cell and record-ing of the mirrored signal using a confocal microscope. Reproduced with permission from [138]. (**c**) The charge distribution in an EDL based FET biosensor with the absence of a cell in the left panel and presence of the cell in the right panel Reproduced with permission from [141].

Luo et al. [139] demonstrated the significant strides made in EEG monitoring via EDL-gated FET biosensors, introducing a wearable, non-invasive methodology for capturing cerebral activity. The employment of these devices in sleep research, neurofeedback therapy, and the diagnosis and treatment of neurological conditions such as epilepsy, Alzheimer’s disease, and Parkinson’s disease is noteworthy. The research underscored high accuracy in differentiating various mental states when these devices were employed to detect cerebral activity during diverse cognitive tasks. Additionally, Luo et al. [139] highlighted how EDL-gated FET biosensors have significantly contributed to EMG monitoring. These devices enable the measurement of EMG signals, mirroring the electrical activity of muscles. These measurements can be used to assess muscle function, diagnose neuromuscular disorders, and guide rehabilitation efforts. Reports of these systems being used to monitor muscle activity in amputees and control robotic prostheses, as stated in recent research [140], illustrate potential applications. Moreover, comprehensive EMG systems capable of measuring muscle activity and incorporating flexible strain and temperature sensors have been developed. This system provides a wealth of information about muscle function and health, as depicted in Figure 12a.

Beyond EEG and EMG monitoring, EDL-gated FET biosensors have significantly contributed to the detection of cellular transmembrane potential, specifically in the context of ion channels and bioelectric signals in cells. Moreddu et al. introduced a novel approach offering a non-invasive and high-throughput method for monitoring bioelectrical activity, as shown in Figure 12b [138]. Their research revealed that the presence of a HEK-293 cell on the electrode increased fluorescence intensity output by 5.8% compared to bare microelectrodes. This technology holds the potential for evaluating cell-substrate adhesion and monitoring cell proliferation, opening new possibilities for examining the electrical phenomena involved in cell migration and cancer progression. Pulikkathodi et al. presented an EDL-gated FET-based biosensor technology capable of detecting biomarkers [141], such as whole cells and transmembrane potential, with high sensitivity. This sensor can record the bioelectric signals of cells and assist in the diagnosis and prognosis of diseases like cancer by identifying specific cell types and functions. The principle of their work is depicted in Figure 12c.

The EDL-gated FET biosensor array, therefore, not only serves to monitor bioelectrical signals like EEG and EMG but also carries significant potential in applications involving cellular bioelectricity. This technology, with its capacity to detect changes in cellular transmembrane potential, can be utilized for studying cellular activities and responses, thus potentially contributing to advancements in diagnostics and therapeutics.

### 5.2. Therapeutic Applications of Epidermal Electronics

In the therapeutic domain, epidermal electronics play a crucial role. They are deployed to monitor treatment effectiveness and optimize treatment strategies. Their applications span robotics and prosthetics, healthcare and fitness, and rehabilitation.

#### 5.2.1. Therapeutic Applications in Robotics and Prosthetics

EDL pressure sensors can be amalgamated into robotic and prosthetic limbs to furnish precise tactile feedback and control, thereby aiding therapy and the rehabilitation process. Xu et al. [142] developed a stretchable and anti-impact iontronic pressure sensor featuring an ultra-broad linear range for biophysical monitoring and deep learning-aided knee rehabilitation. The sensor can track biophysical signals like pulse waves, muscle movements, and plantar pressure, while accurately predicting knee joint postures for improved rehabilitation post-orthopedic surgery.

#### 5.2.2. Therapeutic Applications in Healthcare and Fitness

**Figure 13:**
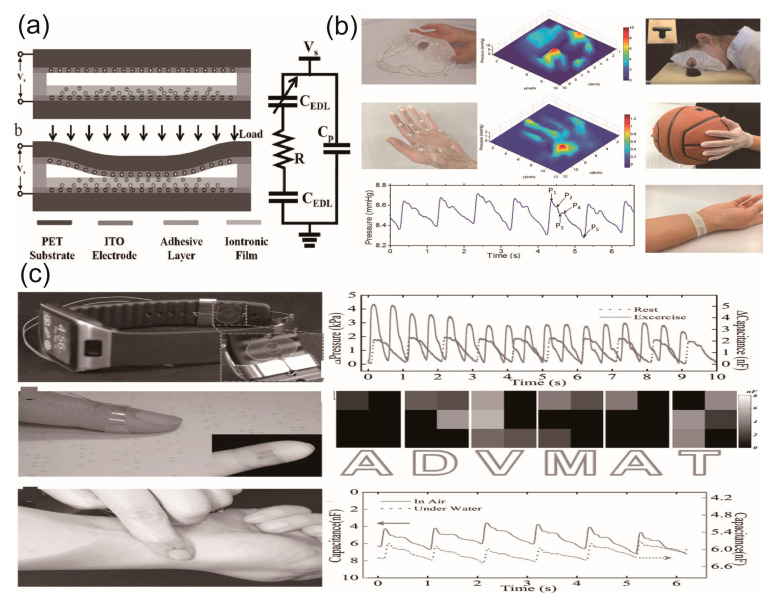
Demonstration of Electrochemical sensors [133,143] (**a**) The working principle of an ionic gel pressure sensor. Reproduced with permission from [133]. (**b**) This image presents the experimental setup used to demonstrate a sensorized robotic fingertip on a soft hand, which is controlled by a robotic arm. This setup is designed to touch, apply, and measure pressure to and from a human arm. Reproduced with permission from [143]. (**c**) The iontronic film with applications in the context of Braille textbook to enhance accessibility for visually impaired individuals and underwater pressure sensor. Reproduced with permis-sion from [133].

In healthcare and fitness, EDL pressure sensors deliver real-time feedback on body movement, posture, and muscle activation patterns. This data can help prevent injuries, enhance athletic performance, and aid in the development of customized exercise routines, thus serving therapeutic purposes. Nie et al. [143] introduced a flexible, transparent iontronic film serving as a thin-film capacitive sensing material for wearable and health-monitoring applications. The film can measure pressure distribution and dynamic pressure changes with superior resolution and accuracy, as shown in Figure 13a,b. Figure 11c demonstrates one such application.

#### 5.2.3. Therapeutic Applications in Rehabilitation

EDL pressure sensors can support physical therapy and rehabilitation by tracking muscle and bone movements, identifying potential complications as summarized by Table 4. These results can guide clinicians in providing effective and personalized treatment for patients. For example, Xu et al. [142] reported the use of EDL pressure sensors for monitoring knee joint posture and movement post-orthopedic surgery. The collected data can be processed through deep learning algorithms to provide feedback and guidance for improved rehabilitation.

Furthermore, EDL-based epidermal electronics can also play a role in drug delivery. Kim et al. [86] presented research utilizing the electric double-layer effect to precisely control the permeability of nanopores and regulate molecular flow. The researchers designed an electrically gated nanoporous membrane composed of metal oxide nanotubes coated with polymers possessing different charges inside and outside the tubes. Applying an electric field caused the polymers to either repel or attract, changing the pore size and dynamically regulating the passage of molecules. The research demonstrated that this electrically gated nanoporous membrane could control drug molecule delivery precisely. The flow of drugs could be swiftly turned on or off by merely altering the electric field parameters, highlighting promising applications of electronic devices in drug therapy and controlled release.

Despite the remarkable progress made for EDL sensors in healthcare, a number of potential challenges and limitations need to be considered. Safety and biocompatibility are paramount for biomedical devices. Long-term exposure to these devices and any potential side effects should be investigated. For instance, potential allergic reactions to the materials used, the risk of infection from prolonged use, and the potential impact on skin health need to be studied [83,84]. Another major issue lies in the accuracy and reliability of these sensors. While EDL sensors demonstrate high sensitivity and range, factors such as ambient temperature, humidity, and external mechanical stress could potentially influence their performance. As such, calibration protocols and error correction methods should be established to ensure the accuracy and reliability of measurements [77,85]. Finally, while the use of EDL sensors in drug delivery is promising, the control of drug release based on electrical fields is a complex task. Factors such as the rate of drug release, drug degradation, and the effect of electrical fields on drug stability require careful consideration [86].

## 6. Conclusions

In conclusion, the emergence of epidermal electronics holds transformative potential in realms such as healthcare and human-machine interfaces. This paper presents a comprehensive review of the latest developments in EDL-based epidermal electronics, with a particular emphasis on the synthesis and preparation of materials, device fabrication methodologies, and systems-level applications. These advancements have heralded the inception of flexible, conformal, and biocompatible electronic devices that integrate seamlessly with human skin.

However, despite these noteworthy achievements, significant challenges remain that limit the potential enhancement of EDL-based devices, specifically the biocompatibility, stability, reliability, and scalability. The biocompatibility of these devices is challenged by the materials, which can induce skin irritation, inflammation, or hypersensitivity reactions, and potential electrolyte leakage or evaporation could pose hazards to both the skin and the environment. The stability of EDL-based devices is vulnerable to external factors such as mechanical deformation, temperature fluctuations, humidity alterations, and sweat secretion. Furthermore, the reliability of these devices can be influenced by several factors like electrode surface roughness, electrolyte viscosity, ion size, valence, concentration, and applied voltage, which may lead to variations in device capacitance or impedance, introducing potential errors or noise into signal detection or transmission. Lastly, the scalability of these devices faces restrictions due to the limitations of traditional fabrication methods, such as photolithography, inkjet printing, and screen printing, and the complexity increases with the size or number of devices, necessitating sophisticated design and fabrication approaches.

## Figures and Tables

**Table 2:** Design of EDL materials for diagnostics and drug delivery applications.

Purpose	Materials	Outcomes	Reference
Diagnostic	ZnO	Detecting Cortisol in Sweat	[83]
	silicon nanowires, carbon nanotubes and graphene	Mechanisms for detecting biomolecules using transistors are described	[84]
	field-effect transistor (FET),monoclonal antibody, saliva collection device	detect SARS-CoV-2 nucleocapsid protein	[50]
	Gold leaf, silver ink pens, double-sided tape, plastic transparencies and fabric adhesives	Enables detection of hydrogen peroxide and glucose	[85]
Drug delivery	Anodized aluminum oxide film, Cr-Au-Cr layer	Realization of intelligent molecular flow control using field effect gating to achieve regulation of drug delivery rates	[86]

**Table 3:** Comparison of fabrication methods.

Methods	Materials	Resolution	Speed	References
**Lithography**	Silicon, glass, polymers, metals	Sub-micron to nanometer scale	High for large batches, low for small batches.	[87]
**Laser cutting**	Metals, wood, acrylic, leather, paper	around 0.1 mm.	High for thin materials, low for thick materials.	[63,89,90]
**Inkjet printing**	polymers, metals, ceramics, biomolecules	around 10 to 50 microns	Moderate to high depending on the printer speed and substrate size.	[91]
**Screen printing**	graphene, metal, carbon-based inks	around 25 to 100 microns	Low to moderate depending on the number of colors and layers.	[92]
**3D printing**	Plastics, metals, ceramics, composites	around 50 to 300 microns	Low to moderate depending on the technology and part size.	[94,95,96]
**Aerosol jet printing**	conductors, semiconductors, insulators	around 10 microns	Low to moderate depending on the nozzle speed and substrate size.	[97,98,99]
**Transfer printing**	inorganic semiconductor metal materials	around 50 to 100 microns	Moderate to high depending on the heat and pressure settings and substrate size.	[100,101,102,103]

**Table 4:** EDL-Based Sensors and Electronics Applications.

Device	Diagnostic	Therapeutic	References
**Pressure Sensors**	Human muscle movement, body movement, posture, muscle activation patterns, pulse waves, and blood pressure, prosthetic and robotic limbs for tactile feedback and control	Assist in therapy and rehabilitation by providing tactile feedback and control for prosthetic and robotic limbs Provide real-time feedback on body movement, posture, and muscle activation patterns for healthcare and fitness Track muscle and bone movements and guide personalized treatments for rehabilitation	[127,128,129,130,133,134,138,139,141,144]
**Bioelectricity**	Ion channels and bioelectric signals	Control the permeability of nanopores and regulate molecular flow for drug delivery Monitor the Bioelectric signal for healthcare and rehabilitation	[86,142,143]

## Data Availability

Data sharing not applicable.
